# Ageing attenuates bone healing by mesenchymal stem cells in a microribbon hydrogel with a murine long bone critical-size defect model

**DOI:** 10.1186/s12979-022-00272-1

**Published:** 2022-03-12

**Authors:** Hirohito Hirata, Ning Zhang, Masaya Ueno, Danial Barati, Junichi Kushioka, Huaishuang Shen, Masanori Tsubosaka, Masakazu Toya, Tzuhua Lin, Ejun Huang, Zhenyu Yao, Joy Y. Wu, Stefan Zwingenberger, Fan Yang, Stuart B. Goodman

**Affiliations:** 1 Department of Orthopaedic Surgery, Stanford University, Stanford, California USA; 2 Department of Orthopaedic Surgery, Saga University, Saga, Japan; 3 Department of Medicine, Stanford University, Stanford, California USA; 4 University Center for Orthopaedics, Traumatology, and Plastic Surgery, University Hospital Carl Gustav Carus at Technische Universität Dresden, Dresden, Germany; 5 Department of Bioengineering, Stanford University, Stanford, California USA

**Keywords:** Ageing, Bone healing, interleukin-4, Mesenchymal stem cell, Microribbon hydrogel

## Abstract

**Background:**

Despite the high incidence of fractures and pseudoarthrosis in the aged population, a potential role for the use of mesenchymal stem cells (MSCs) in the treatment of bone defects in elderly patients has not been elucidated. Inflammation and the innate immune system, including macrophages, play crucial roles in the differentiation and activation of MSCs. We have developed lentivirus-transduced interleukin 4 (IL4) over-expressing MSCs (IL4-MSCs) to polarize macrophages to an M2 phenotype to promote bone healing in an established young murine critical size bone defect model. In the current study, we explore the potential of IL4-MSCs in aged mice.

**Methods:**

A 2 mm femoral diaphyseal bone defect was created and fixed with an external fixation device in 15- to 17-month-old male and female BALB/c mice. Microribbon (µRB) scaffolds (Sc) with or without encapsulation of MSCs were implanted in the defect sites. Accordingly, the mice were divided into three treatment groups: Sc-only, Sc + MSCs, and Sc + IL4-MSCs. Mice were euthanized six weeks after the surgery; subsequently, MicroCT (µCT), histochemical and immunohistochemical analyses were performed.

**Results:**

µCT analysis revealed that bone formation was markedly enhanced in the IL4-MSC group. Compared with the Sc-only, the amount of new bone increased in the Sc + MSCs and Sc + IL4-MSC groups. However, no bridging of bone was observed in all groups. H&E staining showed fibrous tissue within the defect in all groups. Alkaline phosphatase (ALP) staining was increased in the Sc + IL4-MSC group. The Sc + IL4-MSCs group showed a decrease in the number of M1 macrophages and an increase in the number of M2 macrophages, with a significant increase in the M2/M1 ratio.

**Discussion:**

IL4 promotes macrophage polarization to an M2 phenotype, facilitating osteogenesis and vasculogenesis. The addition of IL4-MSCs in the µRB scaffold polarized macrophages to an M2 phenotype and increased bone formation; however, complete bone bridging was not observed in any specimens. These results suggest that IL4-MSCs are insufficient to heal a critical size bone defect in aged mice, as opposed to younger animals. Additional therapeutic strategies are needed in this challenging clinical scenario.

## Introduction

Ageing has a negative impact on bone healing and is a risk factor for delayed union and non-union [1]. In addition to the high prevalence of fractures in the elderly, non-union and delayed union are major causes of posttraumatic morbidity and mortality [1, 2]. Therefore, improving treatment methods to promote bone healing in the elderly remains an urgent issue and unmet clinical need.

One promising treatment is the use of mesenchymal stem cells (MSCs) to treat fractures and has attracted much attention. Researches have been reported on the local implantation of MSCs to promote bone healing[3–5]. However, ageing induces various detrimental effects in MSCs and the host [6–8]. Few studies have examined the efficacy of MSC-based therapies in fracture models in aged animals.

Previously, we developed a therapeutic method of introducing MSCs into bone defect sites by mixing the MSCs with a gelatin microribbon (µRB)-based scaffold (Sc). This scaffold is porous and provides structural support without inhibiting inter-cross links between cells. The Sc facilitates efficient stem cell delivery and cell survival [9–12]. Furthermore, in a cranial bone defect mouse model, MSCs delivered with µRB-Sc promoted vascular ingrowth, chondrogenesis, and osteogenesis more than MSCs alone [9–12].

Other factors affecting MSCs in bone healing must be considered. The immune system, including macrophages, is essential for fracture healing [12–15]. The hematoma produced by bone injury initiates an inflammatory response, cell migration and intercellular cross-talk [16]. The pro-inflammatory milieu including a variety of cytokines such as interleukin (IL)1, IL6, and tumour necrosis factor (TNF)-alpha induces naïve M0 macrophages to polarize into an M1 pro-inflammatory phenotype, leading to the release of cytokines and chemokines, which are necessary for bone healing to progress [12]. Concurrent with inflammation, macrophages also secrete growth factors and chemokines such as transforming growth factor-beta (TGFβ) and insulin-like growth factor (IGF), which are critical during the inflammatory phase of bone healing [15, 16]. After the inflammation subsides at the appropriate time, the repair phase begins. Macrophages polarize to an M2 phenotype and contribute to tissue repair [17].

It has been reported that the polarization of macrophages to an M2 phenotype can be promoted by IL4 and IL13 [18–20]. We have created IL4 over-expressing MSCs (IL4-MSCs) using lentiviral vectors for sustained strong expression of IL4 [20]. Furthermore, consistent with the fact that a period of acute inflammation is required for healing after fracture, we found that suppressing inflammation at 72 h after injury is optimal for accelerating fracture healing [21]. Therefore, in our an established femoral diaphyseal critical size bone defect model [22], IL4-MSCs within µRB scaffolds were implanted at 3 days after primary surgeries. In young male and young female mice, treatment with MSCs within Sc facilitated bone healing in this model; IL4-MSCs also robustly facilitated bone formation by promoting polarization to M2 macrophages [23, 24].

M1 macrophages from elderly mice have higher resting TNF-alpha expression levels than those from young mice and M1 macrophages respond more strongly to inflammatory stimuli and express higher levels of cytokines than young mice [6]. These factors may contribute to prolonged inflammation and increased incidence of delayed fracture healing in the elderly. In the current study, we explore the potential anti-inflammatory effects of IL4-MSCs for facilitating the healing of bone defects in aged mice.

## Materials and methods

### Animals

 This animal experiment protocol was approved by the Institutional Administration Panel for Laboratory Animal Care at Stanford University (Protocol number: 26,905). Institutional Guidelines for the Care and Use of Laboratory Animals were observed in all aspects of this project. We used 15- to 17-months-old BALB/c male and female mice (Jackson Laboratory, Bar Harbor, ME, United States). All the animals were kept on a 12-h light-and-dark cycle and fed a standard diet with food and water ad libitum.

### MSCs; isolation and manipulation

MSCs derived from bone marrow for each sex were isolated according to a previously published method [25, 26]. In brief, we collected bone marrow from both femurs and tibias of 8- to 10-week-old BALB/c male and female mice. Then we suspended bone marrow carefully and filtered through a 70 μm strainer, spun down, and resuspended them in alpha- minimal essential medium (α-MEM, Thermo Fisher Scientific, Waltham, MA, United States) supplied with 10% fetal bovine serum (FBS, Invitrogen, Carlsbad, CA, United States) and antibiotic antimycotic solution (100 units of penicillin, 100 µg of streptomycin and 0.25 µg of Amphotericin B/ml; Hyclone, Thermo Fisher Scientific, Waltham, MA, United States). The unattached cells were removed by replacing the culture media after 24 h, and the remaining adherent cells were defined as passage one. The immunophenotype of isolated MSCs according to International Society for Cell Therapy (ISCR) [27] (CD105+ /CD73+ /CD90.2+ /Scal + CD45−/ CD34 − CD11b−) was characterized by flow cytometry (LSR II, Stanford Shared FACS Facility, Stanford, CA, United States) at passage four. In the current study, we used MSCs between passages four to eight. We produced genetically modified MSCs that over-express IL4 by infecting MSCs with the lentiviral vector carrying murine IL4 gene based on our previous protocol [23]. Briefly, the recombinant lentivirus was produced in HEK293T cells by co-transfecting with the transfer plasmid (pCDH-CMV-mIL-4-EF1-copGFP), packaged plasmid (psPAX2), and enveloped plasmid (pMD2G VSVG) using a calcium phosphate transfection kit (Takara Bio United States Inc., Mountain View, CA, United States) with 25mmol/L chloroquine. We collected and detected the titer of the supernatants of the culture media containing viruses 48 h after transfection. The virus was mixed in MSCs culture medium supplemented with 6 µg/ml of polybrene (Sigma Aldrich, St. Louis, MO, United States) with the multiplicity of infection 100 for MSC infection [28–30].

### Gelatin µRB-based scaffold

The fabrication of gelatin µRBs using a wet-spinning process was conducted according to a previous report [11]. Briefly, to form a viscous solution, gelatin was stirred in dimethyl sulfoxide (20 wt%) at 60 °C for 18 h at 60 rpm. Subsequently, the gelatin solution was transferred to a 60 ml syringe and ejected using a syringe pump set to 5 ml/h into ethanol located 1.8 m under the syringe being stirred at 500 rpm. The precipitated gelatin microfiber was transferred to acetone for 3 h to dry and form µRBs. After being transferred to ethanol, the µRBs were chopped to short length using a homogenizer. Next, the µRBs were transferred to methanol containing methacrylic acid N-hydroxysuccinimide ester (15 wt%) and stirred for 18 h at room temperature to functionalize them. Then, the µRBs were transferred to fresh methanol containing glutaraldehyde (0.1 wt%) and stirred vigorously for 18 h at room temperature. The glutaraldehyde was neutralized by adding L-lysine hydrochloride (1% in 200 ml PBS) and stirring for 2 h. The product was washed eight times with PBS and three times with deionized water to remove the reagents. After that, the product was freeze-dried and stored at -20 °C.

To fabricate scaffolds, the µRBs were rehydrated using PBS containing 0.05% LAP photo-initiator. The µRBs were gently mixed with trypsinized MSCs suspended in PBS after incubation for 1 h at 37 °C. The cell concentrations were 10 million cells/mL. The µRBs containing cells were filled to 2 mm diameter cylindrical mold and exposed to ultraviolet light (365 nm, 2 mW/cm2) for 4 min to produce macroporous scaffolds. The scaffolds were then gently pushed out from the mold and kept in PBS for further applications.

After encapsulation, cell properties and cell viabilities were tested and reported previously [9].

### Surgical procedure and postoperative care

**Fig. 1:**
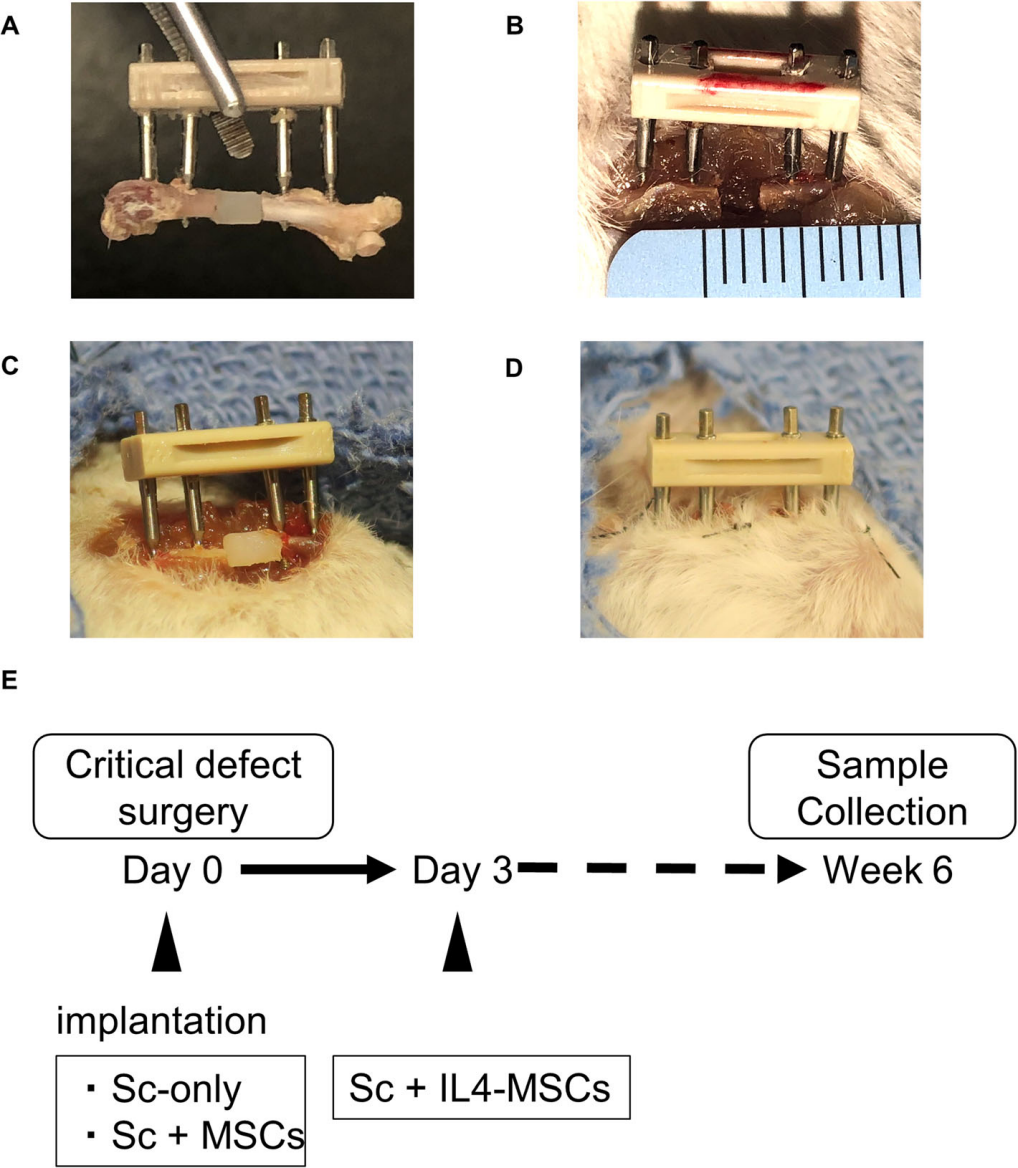
Animal model and surgical procedure and protocol. **A** A model of the external fixator and µRBs. **B** Bone defect of 2 mm. **C** Implantation of a µRBs scaffold with or without MSCs with an external fixator. **D** Postoperative condition of the femur. **E** Surgical protocol. Sc-only and Sc + MSCs groups were implanted on day 0, while the IL4-MSCs group was implanted on day 3 after generation of the surgical defect

All mice received a subcutaneous injection of 0.1 mg/kg buprenorphine for preoperative analgesia before surgery and then performed the surgery. During the operations, mice were anesthetized using inhalation anesthesia with isoflurane in 100% oxygen at a 1 L/ min flow on a warm surgery station for small animals. Two experienced surgeons in each case and one non-operative surgeon assistant conducted the surgery. A 2-mm critical-sized femoral diaphyseal bone defect was made (Fig. 1 A, B), as previously described [22]. Briefly, a longitudinal skin incision was made on the lateral side of the thigh and approached the right femur. After the femur was exposed, a femoral external fixator (MouseExFix, RISystem AG, Landquart, Switzerland) was implanted. Subsequently, a 2 mm critical size bone defect was generated in the femoral midshaft using a Gigli saw. We divided the mouse randomly into three groups for each sex: µRB scaffold without any implantation of cells (Sc-only group), µRB scaffold with unaltered MSCs (Sc + MSCs group), and µRB scaffold with IL4 over-expressing MSCs (Sc + IL4- MSCs group). The cells to be transplanted were designed to be of the same sex as the donor and recipient.

For the Sc-only group and Sc + MSCs group, the surgical procedure and scaffold implantation were performed on the same operative day (Fig. 1 C, D, E).

The scaffolds with IL4-MSCs were implanted 3 days after the primary surgery since the previous in vitro study demonstrated that administration of the anti-inflammatory cytokine IL4 during the first 48 h of culture significantly mitigated acute inflammation, decreased cell proliferation, and downregulated oncostatin M which is recognized to enhance osteogenesis [31, 32]. We closed the surgical incisions with 5–0 Ethilon sutures and injected BuprenorphineSR (0.1 mg/kg) subcutaneously for analgesia after surgery.

### Micro-computational tomography and radiographic analysis

Mice were euthanized by exposure to CO2 followed by cervical dislocation six weeks after the primary surgery. Then samples from each animal were collected. Using a TriFoileXplore CT 120 (TriFoil Imaging, Chatsworth, CA), µCT scans were performed with 50 μm resolution [33, 34]. For the right femur containing the 2 mm critical-size bone defect surgery, we measured the original length of defect based on the µCT images case by case to set the size of ROI (3 mm×3 mm× original length). Next, we calculated the tissue mineral content of the newly formed bone that migrated into the original bone defect area. Finally, the length of the bone defect healing was calculated by this formula: defect healing (mm) = original defect length (mm) - final defect length (mm).

### Histologic analysis and immunohistochemical analysis

The tissue samples were fixed in 4% paraformaldehyde overnight, then decalcified in 0.5 M ethylenediaminetetraacetic acid (EDTA) for 2 weeks, following embedded in optimal cutting temperature compound (OCT) and frozen at -80 °C. Embedded samples were cut into 10 μm-thick sections. For histological analysis, Hematoxylin and Eosin (H&E) staining was performed. According to the scoring system of Huo et al., [35], we evaluate the status of bone healing (Table 1). For Alkaline phosphatase (ALP) staining, we used 1-Step NBT/BCIP Substrate Solution (Thermo Fisher Scientific Rockford, IL, United States). After staining with 1-Step NBT/BCIP Substrate Solution, the ALP- positive area based on the entire area of the scaffold was calculated using the image analysis software program ImageJ (National Institutes of Health, Bethesda, MD, United States) [36].

**Table 1:** The numerical scoring scheme used for the histologic evaluation of fracture healing according to Huo et al.[35].

Score	Associated Finding at Fracture Site
1	Fibrous tissue
2	Predominantly fibrous tissue with small amount of cartilage
3	Equal mixture of fibrous tissue and cartilaginous tissue
4	Predominantly cartilage with small amount of fibrous tissue
5	Cartilage
6	Predominantly cartilage with small amount of immature bone
7	Equal mixture of cartilage and immature bone
8	Predominantly immature bone with small amount of cartilage
9	Union of fracture by immature bone
10	Union of fracture fragments by mature bone

Osteoclast-like cells were stained using a leukocyte tartrate-resistant acid phosphatase (TRAP) staining kit (Sigma Aldrich, St. Louis, MO, United States). Then, we counted TRAP-positive multi-nucleated cells located in the bone defect area. Macrophages were stained and detected as described previously [24]. In brief, the specimens were blocked and permeabilized by 5% BSA with 0.3% Triton X-100 buffer for 60 min at room temperature, followed by primary and secondary antibody incubation. Macrophages were stained by rat anti-CD11b antibody (Abcam, Cambridge, MA, United States) followed by Alexa Fluor® 647 conjugated donkey anti-rat IgG (Abcam, Cambridge, MA, United States). M1 pro-inflammatory macrophages were identified using mouse anti- inducible nitric oxide synthase (iNOS) antibody (Abcam, Cambridge, MA, United States) followed by Alexa Fluor® 488 conjugated goat anti-mouse IgG (Invitrogen, CA, United States). M2 anti-inflammatory macrophages were stained by rabbit anti- liver Arginase (Arg1) antibody (Abcam, Cambridge, MA, United States) followed by Alexa Fluor® 555 conjugated donkey anti-rabbit IgG (Invitrogen, Carlsbad, CA, United States). Slides were mounted by prolong gold antifade mount with DAPI (Life Technologies, Grand Island, NY, United States). Slides were imaged using a fluorescence microscope with 200x magnification (BZ-X800, Keyence, IL, United States). Positive cells in all slides were counted in three randomly selected areas.

### Statistical analysis

Statistical analyses were conducted using GraphPad Prism 8 (GraphPad Software, San Diego, CA, United States). Data are presented as mean ± SE. One-way analysis of variance (ANOVA) followed by the Tukey’s post hoc test was conducted for the multiple statistical comparisons among groups. The difference was considered significant when the p-value was < 0.05.

## Results

### Micro-computed tomography (µCT)

**Fig. 2:**
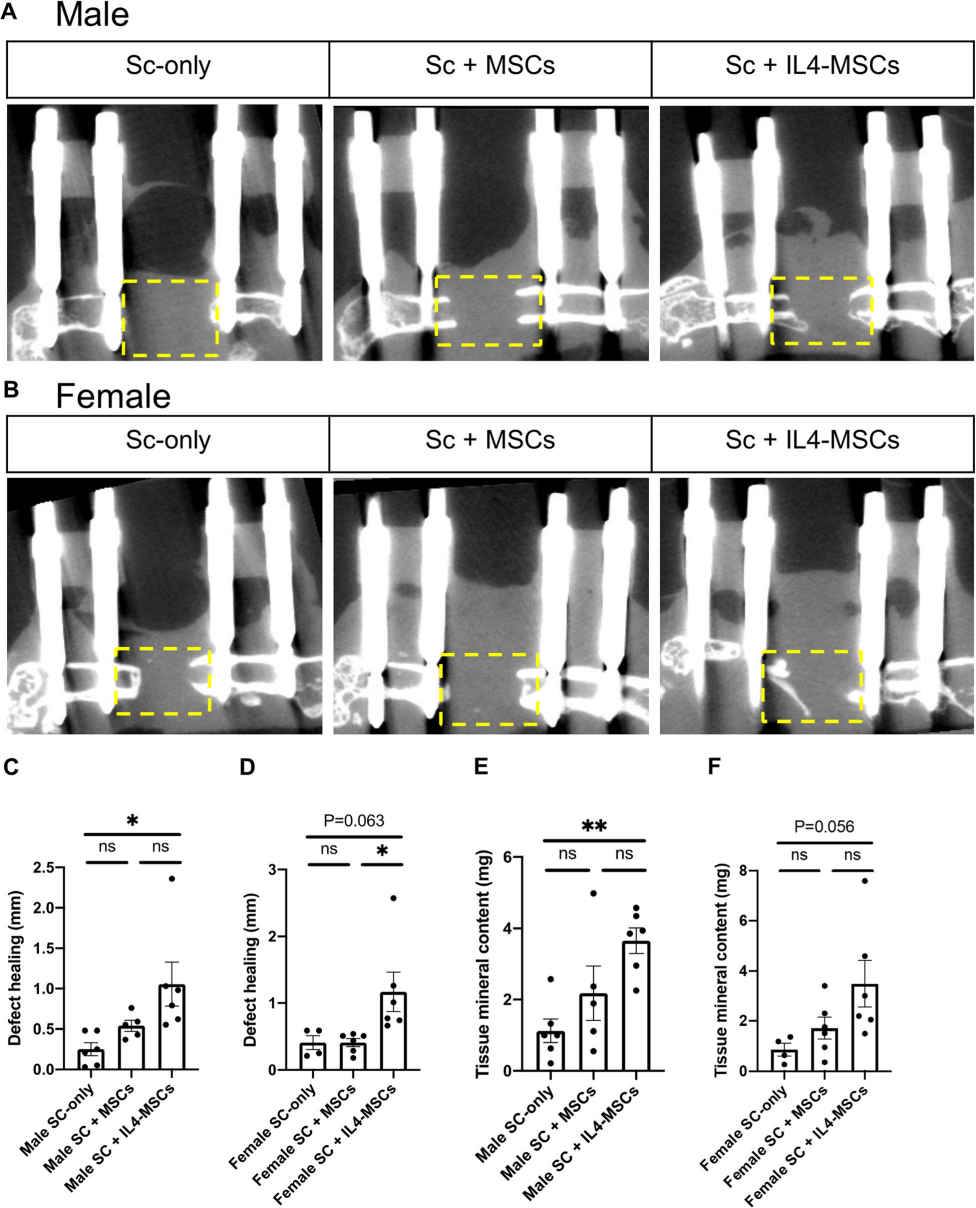
µCT images of the femur in male and female mice. **A** Sagittal view for femurs and defect areas of three groups in Male and (**B**) in Female. **C, D** Defect healing at 6 weeks (mm) and (**E, F**) Tissue mineral content (mg) of the bone defect area were calculated by the µCT. Male Sc-only, *n* = 6; male Sc + MSCs, *n* = 6; male Sc + IL4-MSCs, *n* = 6. Female Sc-only, *n* = 4; female Sc + MSCs, *n* = 6; female Sc + IL4-MSCs, *n* = 6.; *:0.01 ≤ *p* < 0.05, **:0.001 ≤ *p* < 0.01

µCT analysis was performed to determine whether treatment with Sc + MSCs and Sc + IL4-MSCs would promote bone formation after 6 weeks by analyzing the size and tissue mineral content of the bone defect area. First, we measured the defect healing distance (DHD) [24], which reflects new callus formation in the defect. In aged male mice, the Sc + IL4-MSCs group showed a significant increase in DHD compared to the Sc-only group (*p* = 0.014) (Fig. 2 A, C); however there was no significant differences when the Sc + MSCs group was compared to the Sc-only group. In aged female mice, the Sc + IL4-MSCs group demonstrated a strong trend for promoting bone formation (*p* = 0.037) and showed prominent callus formation compared to the Sc + MSCs group (Fig. 2B, D).

Tissue mineral content, which indicates the synthesis of new calcified bone in defect area, increased significantly in the Sc + IL4-MSCs group compared to the Sc-only group in aged male mice (Fig. 2E). The same trend was observed in female mice (*P* = 0.056) (Fig. 2 F). These results indicated IL4-MSCs promote bone formation in aged mice.

### Histological analysis

**Fig. 3:**
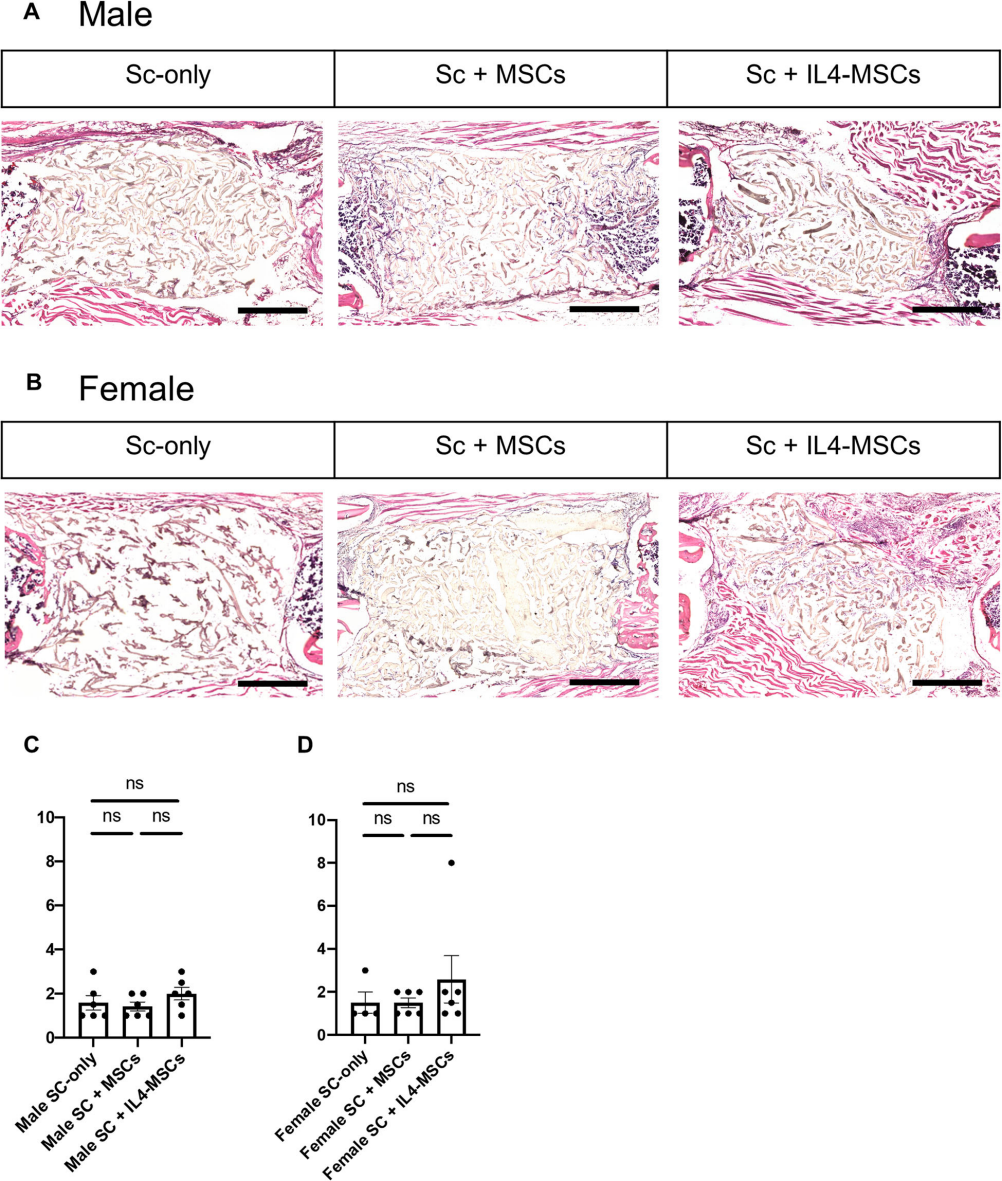
Histological analysis for critical size bone defect healing after 6 weeks in male and female mice. **A** Representative images of H-E staining the bone defect images (scaffolds ± cells) at 200x magnification in male and (**B**) in female. **C, D** Defect healing grade score based on the histological evaluation. Male Sc-only, *n* = 6; male Sc + MSCs, *n* = 6; male Sc + IL4-MSCs, *n* = 6. Female Sc-only, *n* = 4; female Sc + MSCs, *n* = 6; female Sc + IL4-MSCs, *n* = 6

We then performed H&E staining for histological evaluation and quantified bone formation using the method of Huo et al. (Table 1) [35]. Using this method of evaluation, there was no significant differences among the 3 groups (Fig. 3 C, D). Histological analysis demonstrated that the tissues in the defect were primarily fibrous tissue with little evidence of bone or cartilage at 6 weeks. (Fig. 3 A, B).

### Expression of alkaline phosphatase

**Fig. 4:**
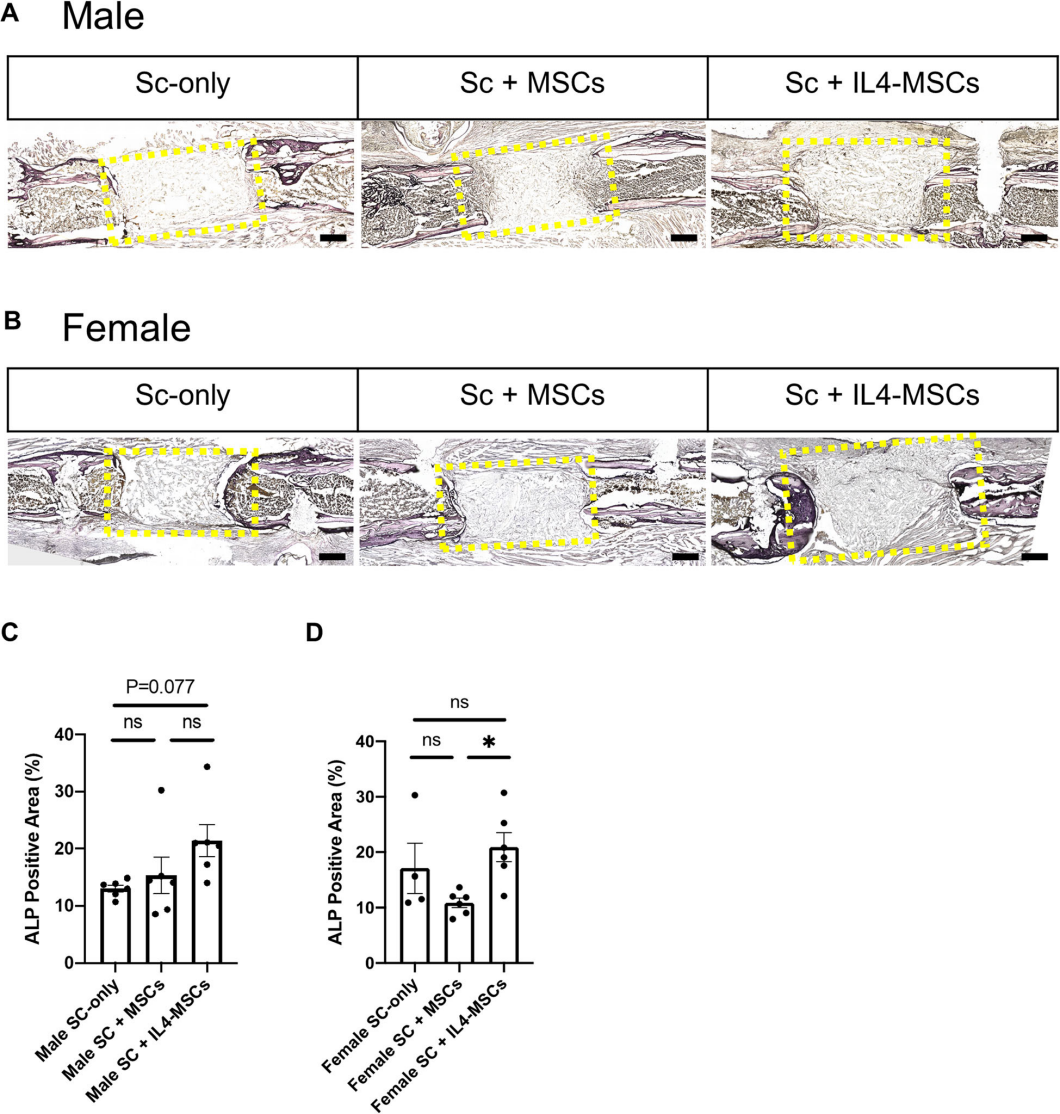
ALP staining and analysis of critical size bone defect healing in male and female mice. **A** The ALP-stained bone defects representative images of all groups after 6 weeks with 200 times magnification in male and (**B**) in female. **C, D** ALP positive area calculated. Male Sc-only, *n* = 6; male Sc + MSCs, *n* = 6; male Sc + IL4-MSCs, *n* = 6. Female Sc-only, *n* = 4; female Sc + MSCs, *n* = 6; female Sc + IL4-MSCs, *n* = 6.; *: 0.01 ≤ *p* < 0.05

Next, we performed ALP staining to evaluate the activity of osteoblasts in the scaffolds. In males, there was an increasing trend of ALP activity in the Sc + IL4-MSCs group, but this did not reach statistical significance (*P* = 0.077) (Fig. 4 A, C). In females, the ALP activity was significantly increased in the Sc + IL4-MSCs group compared to the Sc + MSCs group (Fig. 4B, D).

### Osteoclast formation and activity

**Fig. 5:**
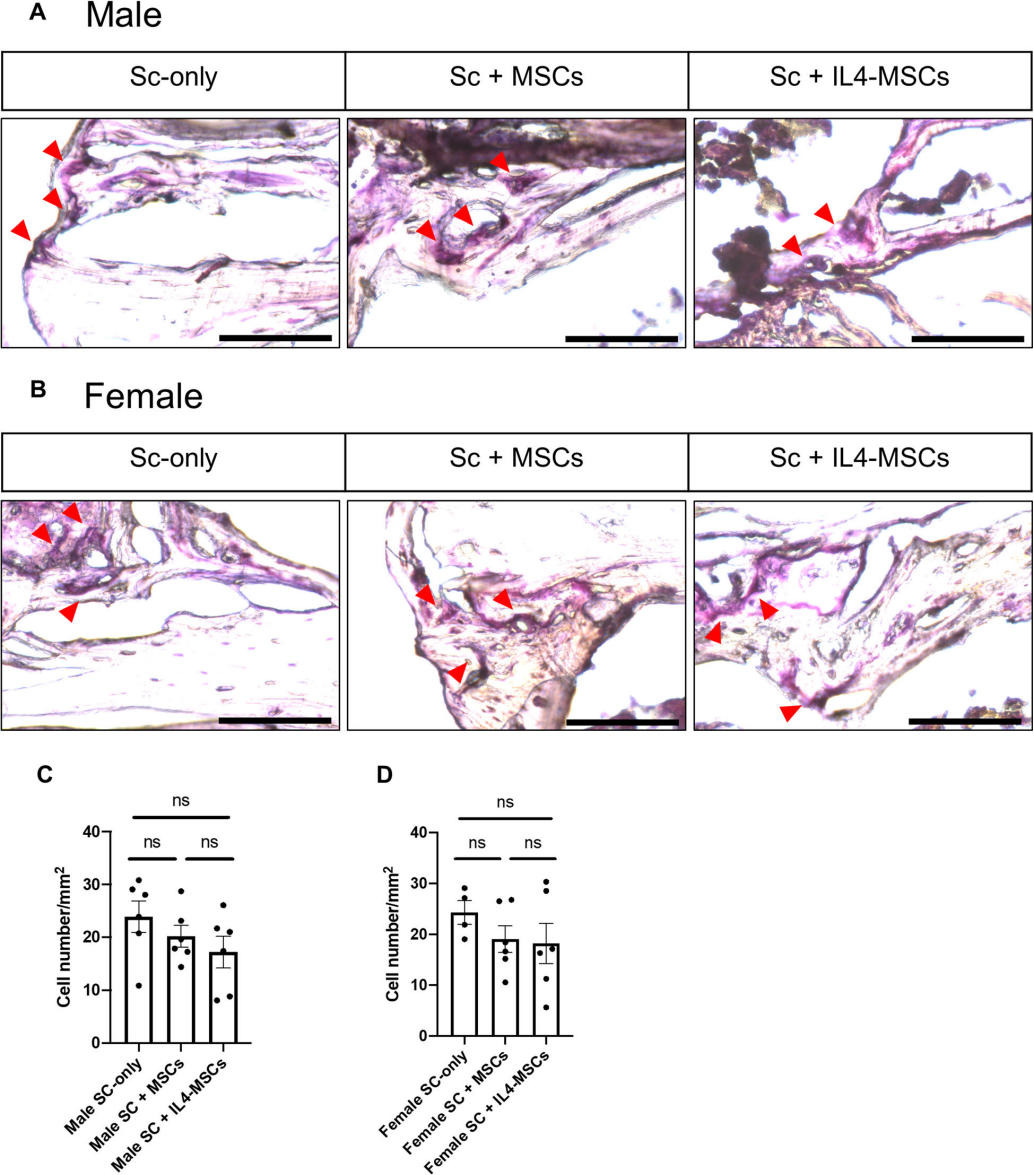
TRAP staining and analysis of critical size bone defect healing after 6 weeks in male and female mice. **A** The TRAP-stained bone defects representative images of all groups with 200 times magnification (red arrows showed the TRAP-positive cells, bar = 100 μm) in male and (**B**) in female. **C, D** TRAP-positive cell number calculated per µm2. Male Sc-only, *n* = 6; male Sc + MSCs, *n* = 6; male Sc + IL4-MSCs, *n* = 6. Female Sc-only, *n* = 4; female Sc + MSCs, *n* = 6; female Sc + IL4-MSCs, *n* = 6

MSCs and IL4 are known to inhibit osteoclast differentiation and activation. We counted the number of TRAP-positive osteoclasts to investigate the effects of the different treatments in the bone defect area. Compared to the Sc-only group, the average number of osteoclasts tended to decrease gradually in the Sc + MSCs group and the Sc + IL4-MSCs group but there was no significant difference (male; *p* = 0.60, 0.22, female; *p* = 0.54, 0.45) (Fig. 5 A-D). These results indicated that inhibition of bone resorption is unlikely to have contributed to the increased bone formation.

### Immunohistochemistry for macrophage and its phenotype

**Fig. 6:**
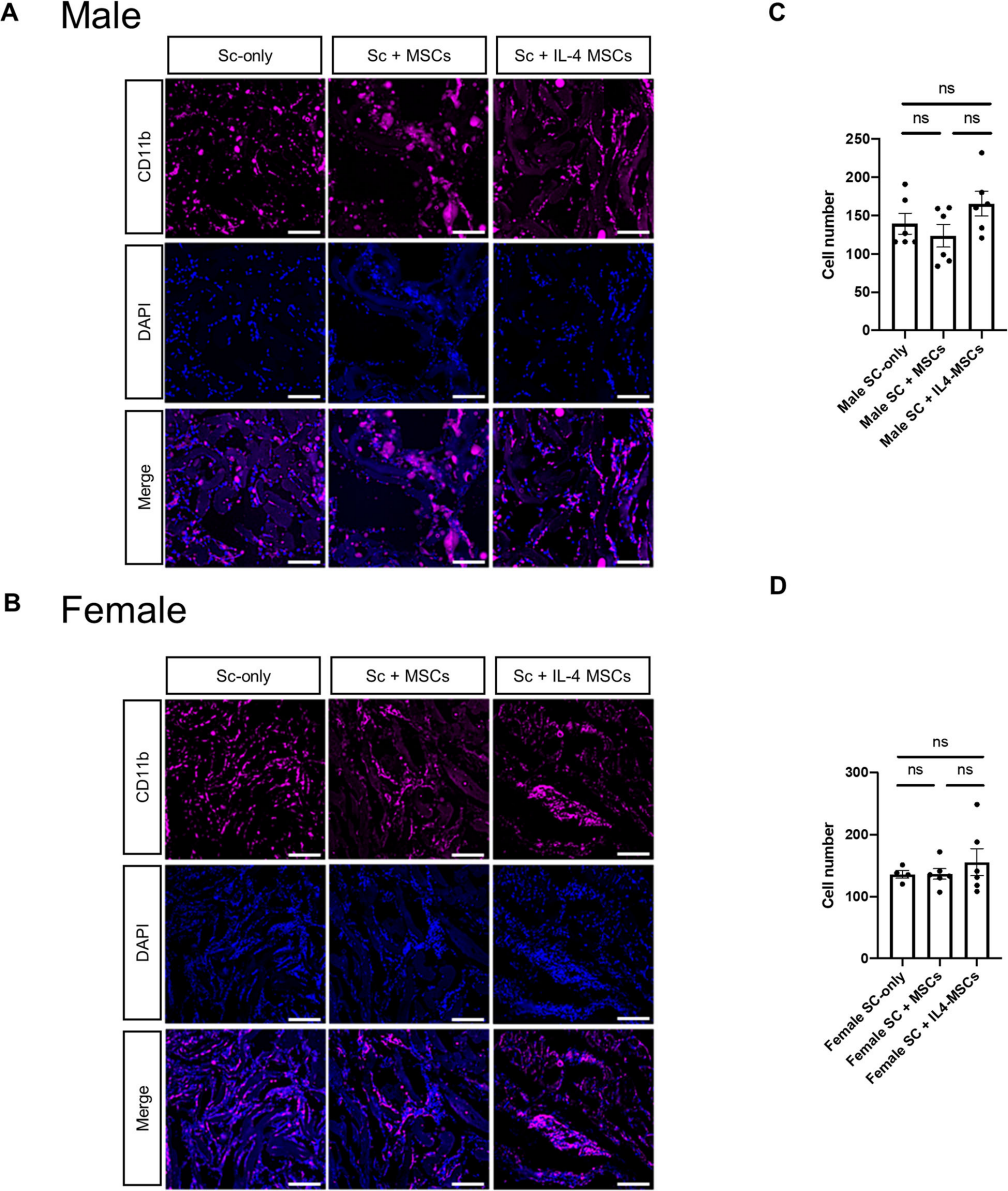
Immunohistochemistry staining and analysis of CD11b/DAPI positive cells inside the scaffolds in male and female mice. **A** Representative images of CD11b/DAPI stained macrophages in all groups (Blue: DAPI/nucleus; Magenta: CD11b/Macrophage marker. Bar = 100 μm) in male and (**B**) in female. **C, D** Number of cells counted from the immunohistochemistry images. Male Sc-only, *n* = 6; male Sc + MSCs, *n* = 6; male Sc + IL4-MSCs, *n* = 6. Female Sc-only, *n* = 4; female Sc + MSCs, *n* = 6; female Sc + IL4-MSCs, *n* = 6

**Fig. 7:**
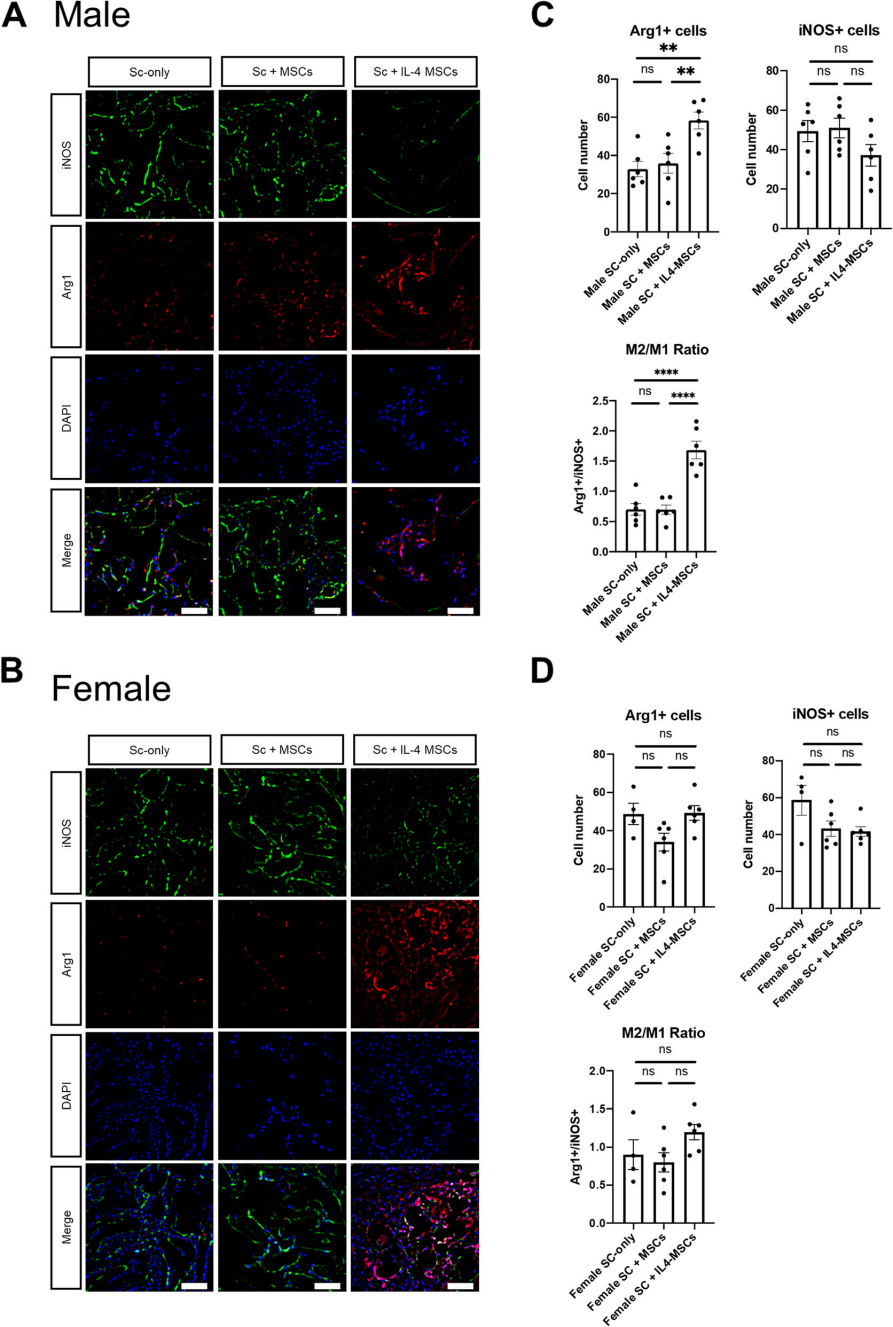
Immunohistochemistry staining and analysis of iNOS/Arg1/DAPI inside the scaffolds in male and female mice. **A** Representative images of iNOS/Arg1/DAPI stained macrophages in all groups (Blue: DAPI/nucleus; Green: iNOS/M1 macrophage marker; Red: Arg-1/ M2 macrophage marker. Bar = 50 μm) in Male and (**B**) in female. **C, D** Number of cells counted from the immunohistochemistry images. Male Sc-only, *n* = 6; male Sc + MSCs, *n* = 6; male Sc + IL4-MSCs, *n* = 6. Female Sc-only, *n* = 4; female Sc + MSCs, *n* = 6; female Sc + IL4-MSCs, *n* = 6.; **:0.001 ≤ *p* < 0.01,****:*p* < 0.0001

We have previously reported that IL4-MSCs promote macrophage migration [23, 24]; thus we measured the number of macrophages that had migrated into scaffolds using immunofluorescence (Fig. 6 A, B). The results revealed no obvious significant differences in the total number of macrophages (CD11b+/DAPI+) among the different groups (Fig. 6 C, D). For the M1 pro-inflammatory macrophage (iNOS+/DAPI+), the number tended to be lower in the Sc + IL4-MSCs group in aged male mice, but this difference was not significant (Fig. 7 A, C). In aged female mice, M1 macrophages tended to decrease in the Sc + MSCs group and the Sc + IL4-MSCs group, but this difference was not significant, either (Fig. 7B, D). The number of M2 macrophages (Arg1+/DAPI+) was markedly elevated in the Sc + IL4-MSCs group in aged male mice (Fig. 7 C), whereas there was no significant difference in the number of M2 macrophages in aged female mice (Fig. 7D). The M2/M1 ratio was evaluated to assess macrophage polarization in the defect [24]. In aged male mice, the M2/M1 ratio was significantly increased (Fig. 7 C); the M2/M1 ratio was also increased in aged female mice, and polarization to M2 was observed, but the differences were not significant due to the variability of data in the control group (Fig. 7D).

## Discussion

The delivery of exogenous MSCs is a promising treatment to facilitate the healing of fractures and bone defects. We have focused on the key interactions between MSCs and macrophages for bone healing using a validated critical size bone defect model. We have previously confirmed the therapeutic effect of IL4-MSC delivery to the bone defect in young male and female mice [24, 25]. However, in clinical practice, fractures are highly prevalent in the elderly, and ageing is an established risk factor for delayed union and non-union [37]. Thus, there is a critical clinical need to develop new strategies for healing of fractures and bone defects in the elderly.

With ageing, inflammatory markers such as IL6 and TNF-alpha are increased [38, 39]. In aged mice, high lipopolysaccharide was observed due to increased intestinal permeability. This condition contributes to an increased low-grade, systemic inflammation in aged mice [40]. Prolonged inflammation is known to delay the healing of fractures [41]. In addition, M1 macrophages demonstrate a disproportionate expression of inflammatory factors such as TNF-alpha to a given stimulus in aged mice, and the normal M2/M1 balance is disrupted [6]. Therefore, we hypothesized that correcting this immune perturbation in the elderly may enhance bone healing. However, in our previous study, we found that prior to 72 h, osteogenesis was depressed by mitigating the stage of acute inflammation [21]. This is a crucial fact in the treatment of fractures. Therefore, we implanted the IL4-MSCs 3 days after surgery.

µCT revealed that osteogenesis was significantly promoted in the Sc + IL4-MSCs group. However, in aged mice, Sc + MSCs did not promote bone healing as robustly compared to that observed in young mice, as reported by various authors [42–46]. In our experiments, we have confirmed that 40% of the transplanted MSCs were still viable after 6 weeks, and they also expressed IL4 [9, 47]. Therefore, the fact that osteogenesis was promoted by Sc + IL4-MSCs but not by Sc + MSCs may be due to immune modulation of the inflammatory environment to a pro-reconstructive milieu. In addition to the immune response, Löffler et al. reported that impaired M2 macrophage function attenuated bone healing in a bone defect model in aged rats. Furthermore, they reported implantation of CD14 + macrophage precursors promoted bone formation [48]. These results support our current findings. However, our aged mice showed incomplete union of the defect.

Various biological properties of cells relevant to the musculoskeletal system alter with age. For example, bone formation is attenuated due to a decrease in osteoblast function, differentiation capacity, cell survival, and secretion of bone matrix [49]. In addition, osteoclast differentiation is accelerated, resulting in a delay in the gain of bone mass and leading to prolonged bone healing[50].

Ageing also increases inflammatory cytokine expression by macrophages and decreases autophagy [51]. On the other hand, the number of macrophages in the bone marrow increases in aged mice, unlike that seen in humans [52]. Furthermore, the angiogenic capacity and anti-inflammatory effects of aged mice macropahges are not lower than those of macrophages in young mice[53], suggesting that environmental changes due to ageing in mice may affect macrophage function more than in humans.

In our previous reports, IL4-MSCs increased macrophage migration into the scaffold and further polarized the macropahges into the M2 phenotype, which markedly promoted osteogenesis [23, 24]. Although polarization of M2 macrophages was observed in the current experiments, contrary to our expectation, migration of macrophages into scaffold was not enhanced in the Sc + IL4-MSCs group. This suggests that the anti-inflammatory effect on the local environment has a limited impact on bone healing in mice, which may have been caused by the ageing of the host systematically and decreased chemokine expression. Furthermore, M2 polarization was observed only in male mice with a significant difference noted, but not in female mice. This result suggests that hormonal effects and subsequent inflammatory responses may vary with sex in aged mice, and be different compared with younger mice.

There are limitations in our experimental model. We used two well-established markers Arg1 and iNOS to identify M2 and M1 macrophages respectively. However, the relative expression of these markers may be age-dependent. In other words, the results observed in younger mice may differ from those in aged mice, despite a similar stimulus.

We evaluated the healing process at one time point, i.e., 6 weeks after surgery. Thus we cannot comment on the evolution of the early post-implantation period, when callus formation may have been actively occurring. We did not perform an evaluation of changes in the defect site after a more extended period. However, based on the previous data showing that IL4-MSCs robustly promoted bone healing in young mice at 6 weeks, we believe the results of current experiments indicate that prolonged chronic inflammation and subsequent fibrosis in aged mice despite treatment with IL4-MSCs has lead to persistence of the bone defect. To avoid the potential adverse effects of early IL4 exposure during the first 48 h of bone healing, we implanted the Sc + IL4-MSCs 3 days after primary surgery. Thus, we cannot comment on the hypothetical results if the IL4-MSCs were introduced at day 0, the day of primary surgery.

In conclusion, µRB scaffolds with IL4 over-expressing MSCs improved the healing in aged mice by polarizing macrophage to M2, however, this effect was insufficient to bridge the bone defect. The current study indicates that other strategies are necessary to resolve this unmet clinical need.

## Data Availability

The datasets used and/or analyzed during the current study are available from the corresponding author on reasonable request.
